# Multigene Expression Programming Based Forecasting the Hardened Properties of Sustainable Bagasse Ash Concrete

**DOI:** 10.3390/ma14195659

**Published:** 2021-09-28

**Authors:** Muhammad Nasir Amin, Kaffayatullah Khan, Fahid Aslam, Muhammad Izhar Shah, Muhammad Faisal Javed, Muhammad Ali Musarat, Kseniia Usanova

**Affiliations:** 1 Department of Civil and Environmental Engineering, College of Engineering, King Faisal University (KFU), P.O. Box 380, Al-Hofuf 31982, Saudi Arabia; kkhan@kfu.edu.sa; 2 Department of Civil Engineering, College of Engineering in Al-Kharj, Prince Sattam Bin Abdulaziz University, Al-Kharj 11942, Saudi Arabia; f.aslam@psau.edu.sa; 3 Department of Civil Engineering, COMSATS University Islamabad, Abbottabad Campus, Abbottabad 22060, Pakistan; arbabfaisal@cuiatd.edu.pk; 4 Department of Civil and Environmental Engineering, Universiti Teknologi PETRONAS, Bander Seri Iskandar 32610, Malaysia; muhammad_19000316@utp.edu.my; 5 Department of Construction of Unique Buildings and Constructions, Peter the Great St. Petersburg Polytechnic University, 195291 St. Petersburg, Russia; plml@mail.ru

**Keywords:** multigene expression programming, experimental investigation, multiphysics models, machine learning, agricultural waste, sustainability, cross-validation

## Abstract

The application of multiphysics models and soft computing techniques is gaining enormous attention in the construction sector due to the development of various types of concrete. In this research, an improved form of supervised machine learning, i.e., multigene expression programming (MEP), has been used to propose models for the compressive strength ($f_{c}^{\prime }$), splitting tensile strength ($f_{\mathrm{STS}}$), and flexural strength ($f_{\mathrm{FS}}$) of sustainable bagasse ash concrete (BAC). The training and testing of the proposed models have been accomplished by developing a reliable and comprehensive database from published literature. Concrete specimens with varying proportions of sugarcane bagasse ash (BA), as a partial replacement of cement, were prepared, and the developed models were validated by utilizing the results obtained from the tested BAC. Different statistical tests evaluated the accurateness of the models, and the results were cross-validated employing a k-fold algorithm. The modeling results achieve correlation coefficient (R) and Nash-Sutcliffe efficiency (NSE) above 0.8 each with relative root mean squared error (RRMSE) and objective function (OF) less than 10 and 0.2, respectively. The MEP model leads in providing reliable mathematical expression for the estimation of $f_{c}^{\prime }$, $f_{\mathrm{STS}}$ and $f_{\mathrm{FS}}$ of BA concrete, which can reduce the experimental workload in assessing the strength properties. The study’s findings indicated that MEP-based modeling integrated with experimental testing of BA concrete and further cross-validation is effective in predicting the strength parameters of BA concrete.

## 1. Introduction

The damage caused by the construction industry to the environment is a well-known fact. The construction sector uses a third of the total energy production and emits a large amount of greenhouse gases into the atmosphere [1]. Concrete is a widely used material that emits 0.13 tons of CO_2_ per ton of concrete produced [2,3,4]. The idea of green concrete is gaining popularity as a way to diminish the harmful impacts of concrete while still addressing the underlying problem. Green concrete is made by substituting industrial waste with traditional cementitious materials. Commonly used wastes that can be used as cement replacement are electric arc furnace slag, rubber ash, fly ash, volcanic ash, rice husk ash, metakaolin, and sugarcane bagasse ash [5,6]. The use of these materials is seen as a low-carbon alternative to traditional building materials and a way to reduce energy use and carbon emissions effects. Sugarcane bagasse is the primary fuel used in the sugarcane industry around the world [7]. It is one of the agricultural wastes that remain after processing and extraction in the same industry. Bagasse and residue ash make up about 26% and 0.62% of each ton of sugarcane, respectively [8]. The ash is disposed of in landfills, raising significant environmental problems [9]. As a result, environmentally friendly applications for bagasse ash (BA) are being explored in the construction sector. Several experimental studies have indicated that BA can be used as a cement substitute in concrete with a substantial improvement in mechanical properties. Chusilp et al. (2009) [10] observed that concrete including 20% BA by mass of binder had greater compressive strength ($f_{c}^{\prime }$) and better stability. Sobuz et al. (2014) [11] stated that maximum strength of BA concrete (BAC) could be obtained by replacing 10% of the cement. According to Jagadesh et al. (2018) [12], the $f_{c}^{\prime }$ of the concrete with 30% raw BA was reduced by nearly 50%. The reduction in $f_{c}^{\prime }$ was attributable to the larger size of particles, which expands the pore size. Almost a 27% increase in $f_{c}^{\prime }$ was observed when 10% binder was replaced with BA. The increase in $f_{c}^{\prime }$ is caused by the presence of finer silica and the finer BA particles acts as a filler, which in turn improves the density and strength of concrete. Similar observations are reported by Bahurudeen et al. (2015) [13] where the authors noted that at 25% replacement, the compressive strength of BAC decreases due to dilution effect. In addition, the durability properties of BAC are reported to be much better than normal concrete [10,14,15].

Hence, it can be concluded from the aforementioned discussion that the behavior of BAC is different for different percentages of cement replacement. This complex behavior is due to several factors, including the proportion of concrete used in the mixture, the percentage of cement replacement, the type of aggregates, and different water-to-binder ratios. The development of such relations and factors will increase the use of BAC in the building industry. Recent advances in the area of artificial intelligence (AI) and multiphysics models have brought about substantial changes in many engineering fields, including aeronautical engineering, mechanical engineering, and civil engineering [16,17,18,19]. Materials engineers used advanced soft computing techniques to predict different properties of the materials, including compressive strength ($f_{c}^{\prime }$) and splitting tensile strength.

The applications of the random forest (RF) technique for modeling the compressive strength ($f_{c}^{\prime }$) of high-strength concrete was reported by Farooq et al. (2020) [20]. To examine the $f_{c}^{\prime }$ of synthetic-sand concrete, the RF model was created by Zhang et al. (2020) [21]. The results of the study reported the reduced performance of RF when compared with similar models. Sun et al. (2019) [22] predicted the $f_{c}^{\prime }$ of concrete in which rubber was replaced with fine aggregate. The author combined the RF method with different optimization techniques and reported high accuracy for the combined methods. Chou et al. [23] applied a support vector machine (SVM) to forecast the $f_{c}^{\prime }$ of high-strength concrete with Kernel function for model development. Outcomes of the research indicated a reliable and high prediction accuracy of the SVM model. In another study conducted by Deng et al. (2018) [24], SVM was employed to establish a model for the $f_{c}^{\prime }$ of recycled aggregate concrete. Based on statistical analysis, the SVM showed an acceptable modeling outcome. Alexiadis et al. (2019) [25] coupled a deep multiphysics model with machine learning algorithm in parallel. The authors discussed the practical and theoretical aspects of the particle neuron duality and demonstrated it as an efficient computational method capable to learn during the simulation process. The artificial neural network (ANN) was applied to forecast the $f_{c}^{\prime }$ of rice husk ash concrete, fly ash concrete, lightweight concrete, rubberized concrete, ultra-high strength concrete and modulus of elasticity of concrete made with recycled aggregate [18,26,27,28,29,30,31]. ANN shows reliable performance based on inferential statistics in forecasting the different mechanical properties in these research programs. However, ANN does not provide information of the associated problem, thus meaning it is considered as a black-box model. The ANN models are based on the correlation among the inputs and outputs, though the relation is either linear or dependent on predefined functions [5,32,33]. Recently, gene expression programming (GEP) was employed to estimate the mechanical properties of high strength concrete (HSC), waste foundry sand concrete (WFSC), bagasse ash concrete (BAC), and modeling the bearing capacity of concrete filled steel tubes and RC frame structures [5,34,35,36]. GEP was considered advantageous in terms of providing empirical equations and high prediction capability, and comparative assessment showed better and enhanced accuracy of the GEP. However, the GEP approach was found to have some limitations as it does not take into account a large number of diverging entries for the establishment of model, thus shrinking its range of application [5]. Such outlying entries should be deleted from the GEP model domain to enhance the performance of developed model. Moreover, the GEP encodes just one chromosome, so it is appropriate for the basic connection among the dependent (response) and independent (explanatory) variables [37].

Considering the aforementioned difficulties and limitations, an advanced and improved algorithm, i.e., multigene expression programming (MEP) has been utilized to formulate the mechanical properties of BAC. To the best of the authors’ knowledge, no detailed study has been performed to date to develop a relationship and figure out the responsible factors for the development of strength of BAC using MEP. The MEP has the capability of encoding several chromosomes into a single program (code) and the best possible chromosome can be selected based on evaluating fitness [37,38]. MEP is considered an improved form of the GEP, having the capacity to forecast accurate results given the complexity of the target is unseen compared to other modeling techniques [39]. A simple decoding process is used in MEP as compared to other machine learning (ML) algorithms. Despite the unique attributes of MEP, it has been scarcely utilized in civil engineering. In the present study, the mechanical properties of BAC, such as compressive strength ($f_{c}^{\prime }$), splitting tensile strength ($f_{\mathrm{STS}}$), and flexural strength ($f_{\mathrm{FS}}$) of BA concrete were modeled considering the optimum parameters of MEP to resolve a complex relationship. A large and comprehensive database was extracted from the previously published literature to train the proposed model. After that, the concrete specimen with different dosages of BA was prepared in the lab, and the results of the lab-tested specimen were used to validate and test the established MEP models. The output of the developed models was further cross-validated by the k-fold method. The performance of the final established models was assessed employing several statistical assessment indicators. The robust MEP technique supplemented with experimental tests and statistical checks could effectively solve complex problems.

## 2. Modeling Techniques and Database

### 2.1. Multigene Expression Programming

An improved form of machine learning (ML) known as multigene expression programming (MEP) is recently proposed, in which individual variables are represented by changing length entities [37,40]. The distinguishing feature of MEP is to propose simple linear and numerous solutions in a single chromosome [41]. This unique function enables searching in a broader range to find the finest viable response. Compared to gene expression programming (GEP), the MEP follows simple and easy processes [32]. MEP can handle exceptions such as incorrect expressions, infinity, statistical error type values, etc. As the gene is responsible for generating an exception, it alters to an arbitrarily terminal symbol. Therefore, no infertile individuals enter the next generation, thus providing a margin in the chromosome structure during the assessment and evaluation process. However, the GEP cannot remove such exceptions and may become part of the final solution [37]. The MEP is decoded similarly to the pascal and C compiler empirical relationship to machine coding. The result of the MEP is in a linear string of instructions form [42]. Several genes per chromosome govern the chromosome length, whereas the gene encodes the elements in function and terminal set. The abovementioned advantages of MEP over other methods can lead to accurate and reliable models in many fields. The MEP has been applied in a few research studies to estimate elastic modulus of normal and high strength concrete [41], to formulate the compressive strength of Portland cement [43], to develop models for soil deformation modulus [44], to formulate models for consolidating depth of the soil layer [42] and to develop models for polymer confined concrete columns [39].

The development of MEP model depends on several parameters which affect the overall performance of the model. Therefore, careful selection of these parameters is necessary. The values of the MEP-optimized parameters selected in the present study are presented in Table 1. The trial and error approach was used to get the optimum values of these important parameters, as suggested in the literature [45].

### 2.2. Modeling Database

**Figure 1:**
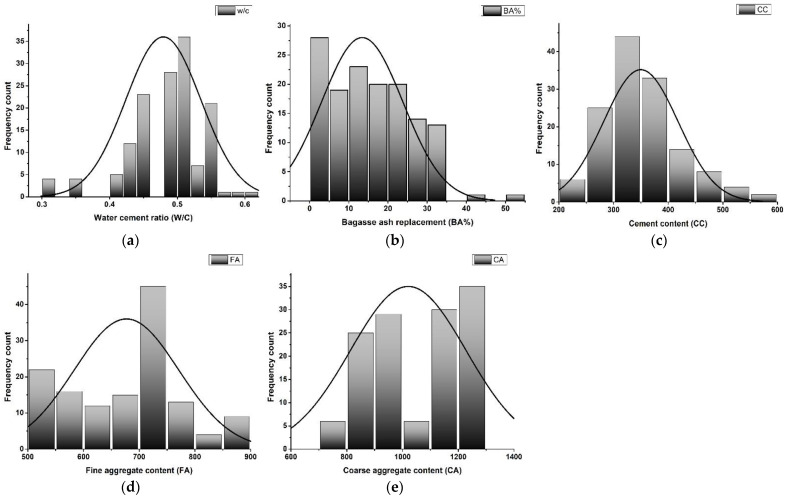
Frequency distribution of the modeling inputs (**a**) w/c, (**b**) BA%, (**c**) CC, (**d**) FA, (**e**) CA.

A comprehensive and reliable experimental dataset on 28 days mechanical properties of bagasse ash concrete (BAC) was acquired from the published literature to train the MEP models [10,11,12,13,14,15,46,47,48,49,50,51,52,53,54,55,56,57,58,59,60,61,62,63,64,65,66,67]. The final datasets included a total of 132, 125, and 128 records of compressive strength $\left(f_{c}^{\prime }\right)$, splitting tensile strength $\left(f_{\mathrm{STS}}\right)$ and flexural strength $\left(f_{\mathrm{FS}}\right),$ respectively, for concrete incorporated with bagasse ash (BA). As some researchers follow the British standard during experimental testing of the compressive strength ($f_{c}^{\prime }$) for concrete, the cube strength data was converted to cylindrical strength to make the data uniform [4,68]. Once all the data was collected and properly arranged, statistical analysis was applied to identify the most important and effective parameters that considerably influence the performance of BAC. The results of the data after statistical analysis are shown in Table 2. The parameters selected in the present research are water-to-binder ratio (w/c), amount of cement (CC), the quantity of coarse aggregate (CA), the quantity of fine aggregate (FA), and the percentage of BA (BA%). The frequency histograms of these modeling inputs are illustrated in Figure 1 for the purpose of visualizing the distribution of the input variables. The aforementioned parameters are considered to be a function of the $f_{c}^{\prime }$, $f_{\mathrm{STS}}$ and $f_{\mathrm{FS}}$ of BAC as given in Equation (1).

(1) $$f_{c}^{\prime },{\;f}_{\mathrm{STS}},{\;f}_{\mathrm{FS}}=f\left(\frac{W}{C},\;\mathrm{SCBA}\%,\;\mathrm{CA},\;\mathrm{CC},\;\mathrm{FA}\right)$$

### 2.3. Cross-Validation with k-Fold Algorithm

The machine learning (ML) models frequently fail to generate generalizable findings when trained on data that has not been previously used for model training. Consequently, it becomes difficult to assess the accuracy of the models [69]. As a usual practice, the dataset is partitioned into train and test sets for training and testing of models, respectively, and the performance is then assessed using statistical error metrics. However, this approach only works well with the availability of a large and broad dataset. Moreover, it is not considered a reliable method as the accuracy of one dataset can be very different from the accuracy obtained for another dataset. A resampling technique, called k-fold cross-validation, is used to ensure that the model can perform well on unseen data. This technique distributes the currently available dataset to k subclasses [70]. The superior results and efficacy of the 10-fold approach are presented in the previously published literature [71]. In the current study, the 10-fold cross-validation is adopted by randomly dividing the dataset into ten subsets. Each class of the 10 subsets is utilized for validation to examine the grouping model, and the same process is reiterated for each subset left behind. The accuracy and predictability of the final model are then expressed in terms of mean accuracy obtained by the 10-fold approach in ten individual rounds.

### 2.4. Models Evaluation by Statistical Measures

Different researchers suggest different parameters to check the accuracy of the developed models. Some of those parameters are used in this study, and their mathematical expressions are presented in Equations (2)–(9). Researchers recently used a new parameter to avoid overfitting of the model in artificial intelligence, and ML, known as an objective function (OF), is also used in this study [41,72]. If the values are low for Equations (2) and (5), the model is said to be good [30]. Similarly, if the values obtained from Equations (3) and (4) are close to 1, the model is termed as good [73]. However, it singlehandedly cannot judge the validity of a model because of its insensitiveness to the multiplication of the division of outcome. Likewise, according to Despotovic et al. (2016) [74], a model is deemed excellent if the result of Equation (7) is between 0 and 0.10; and good if lies between 0.11 and 0.20, respectively. The values of Equations (8) and (9) lie from 0 to positive infinity with a value nearer to zero signifies a good model. Lower value of OF identifies superior model performance.

(2) $$\mathrm{Root}\;\mathrm{means}\;\mathrm{squared}\;\mathrm{error}\;\left(\mathrm{RMSE}\right)=\sqrt{\frac{\sum_{i=1}^{n}{\left(P_{i}-M_{i}\right)}^{2}}{N}}$$

(3) $$\mathrm{Nash}\;\mathrm{Sutcliff}\;\mathrm{efficiency}\;\left(\mathrm{NSE}\right)=1-\frac{\sum_{i=1}^{n}{\left(M_{i}-P_{i}\right)}^{2}}{\sum_{i=1}^{n}{\left(M_{i}-{\bar{M}}_{i}\right)}^{2}}$$

(4) $$\mathrm{Correlation}\;\mathrm{coefficient}\;\left(R\right)=\frac{\sum_{i=1}^{n}\left(M_{i}-{\bar{M}}_{i}\right)(P_{i}-{\bar{P}}_{i})}{\sqrt{\sum_{i=1}^{n}{\left(M_{i}-{\bar{M}}_{i}\right)}^{2}\sum_{i=1}^{n}{\left(P_{i}-{\bar{P}}_{i}\right)}^{2}}}$$

(5) $$\mathrm{Mean}\;\mathrm{absolute}\;\mathrm{error}\;\left(\mathrm{MAE}\right)=\frac{1}{n}\sum_{i=1}^{n}\left|P_{i}-M_{i}\right|$$

(6) $$\mathrm{Relative}\;\mathrm{squared}\;\mathrm{error}\;\left(\mathrm{RSE}\right)=\frac{\sum_{i=1}^{n}{\left(P_{i}-M_{i}\right)}^{2}}{\sum_{i=1}^{n}{\left({\bar{M}}_{i}-{\bar{M}}_{i}\right)}^{2}}$$

(7) $$\mathrm{Relative}\;\mathrm{root}\;\mathrm{mean}\;\mathrm{squared}\;\mathrm{error}\;\left(\mathrm{RRMSE}\right)=\frac{1}{\left|\bar{M}\right|}\sqrt{\frac{\sum_{i=1}^{n}{\left(P_{i}-M_{i}\right)}^{2}}{N}}\;$$

(8) $$\mathrm{Performance}\;\mathrm{index}\;\left(\rho \right)=\frac{\mathrm{RRMSE}}{1+R}$$

(9) $$\mathrm{Objective}\;\mathrm{function}\;\left(\mathrm{OF}\right)=\left(\frac{n_{T}-n_{\mathrm{TE}}}{n}\right)\rho_{T}+2\left(\frac{n_{\mathrm{TE}}}{n}\right)\rho_{\mathrm{TE}}$$

where $n,M_{i},P_{i},{\bar{M}}_{i}{\;\mathrm{and}\;\bar{P}}_{i}$ shows the total number of data points been partitioned into subsets, measured value, predicted value, mean of measured values, and mean of predicted value, respectively of the ith domain. The T and TE are the subscripts that correspond to the train and test datasets, respectively.

## 3. Mix Proportions for Bagasse Ash Concrete (BAC)

A series of experimental tests of bagasse ash concrete (BAC) was completed to validate the behavior of the MEP model through the validation requirement. The modified bagasse ash concrete mixes (BAC) and normal concrete (NC) samples were casted at 25 °C, and cured for 28 days to compare their mechanical properties. Various doses of bagasse ash (BA), ranging from 0% to 40%, were used as a cement replacement. The water-to-cement ratio for all the specimens was kept constant to compare the BAC with NC. Table 3 presents the complete formulation of the mix design proportions. Standard concrete cylinders (300 mm × 150 mm) and beams (100 mm × 100 mm × 500 mm) were produced with varying dosages of BA. The $f_{c}^{\prime }$, $f_{\mathrm{STS}}$, $f_{\mathrm{FS}}$ were tested at 28 days of curing age according to ASTM C39, ASTM C496, and ASTM C293 standards, respectively. The final results of the tested specimens were used to verify the behavior of the MEP models.

## 4. Results and Discussion

### 4.1. Mechanical Properties of BAC

**Figure 2:**
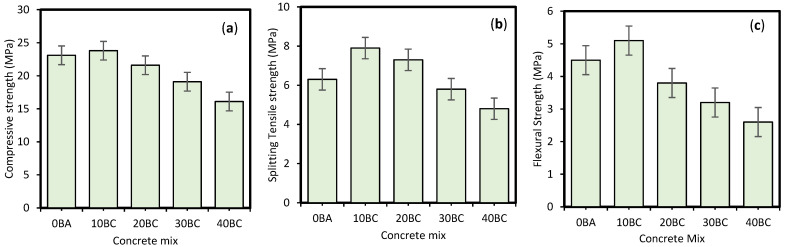
Experimentally tested results of (**a**) compressive strength, (**b**) tensile strength, (**c**) flexural strength of bagasse ash concrete (BAC).

The fundamental mechanical properties of BAC, namely $f_{c}^{\prime }$, $f_{\mathrm{STS}}$ and $f_{\mathrm{FS}}$ were evaluated in the laboratory through testing beams and concrete cylinders using BA from 0 to 40% as a partial cement replacement. It can be noticed from Figure 2 that the strength of concrete increases up to 10BA (10% cement replaced with BA) and consistently decreases for 20BA, 30BA, and 40BA. The maximum strength gained is at 10% cement replacement and may be due to the small finest BA particle dispersed throughout the mix. The silica reacts with lime (resulted from cement hydration) and produces more calcium silicate hydrate (CSH) [49,75]. Additionally, the finer particle size fills the voids and increases the packing density. The strength reduction for higher replacement levels, i.e., 20BA, 30BA, and 40BA, is 6.5%, 17.3%, and 30.3%, respectively. This reduction might be attributable to a lack of sufficient Ca(OH)_2_.

As shown in Figure 2, the maximum $f_{\mathrm{STS}}$ has been achieved by 10BA followed by 20BA. The increase in $f_{\mathrm{STS}}$ relative to NC samples is 25.3% and 15.8% for 10% and 20% substitution of BA, respectively. However, maximum $f_{\mathrm{STS}}$ has been achieved at 10% substitution of BA as a replacement of cement. The $f_{\mathrm{STS}}$ decreases by 7.9% and 23.8% for 30% and 40% BA replacement, respectively. For $f_{\mathrm{FS}}$, the maximum strength is also attained by 10% BA. The increased $f_{\mathrm{STS}}$ and $f_{\mathrm{FS}}$ at 10% BA might be attributed to the micro-fibrous character of BA, associated with CSH production and the generation of aluminates, developing in a needle-shaped structure [76,77]. The interlocking and bonding of such needles occur between hydrated pastes, which immediately enhances $f_{\mathrm{STS}}$ and $f_{\mathrm{FS}}$ of BAC.

### 4.2. Formulation of BAC Mechanical Properties

The MEP findings for $f_{c}^{\prime }$, $f_{\mathrm{STS}}$ and $f_{\mathrm{FS}}$ are evaluated in order to obtain empirical formulations for predicting the abovementioned characteristics related to the five input variables (w/c, BA%, CC, FA, and CA). For $f_{c}^{\prime }$, $f_{\mathrm{STS}}$ and $f_{\mathrm{FS}}$, the resulting MEP formulae are presented as Equations (10)–(12), respectively. Firstly, the essential input parameters were selected based on significant correlation and literature study for the derived equations. The MEP model was then trained on the data acquired from published literature. After acquiring the results predicted by the model, i.e., the RMSE and NSE values, the model is considered to be successfully trained on the given data. At the end of this process, the model provides empirical equations based on the number of input parameters. Finally, the derived Equations (10)–(12), were tested given the testing dataset.

**Figure 3:**
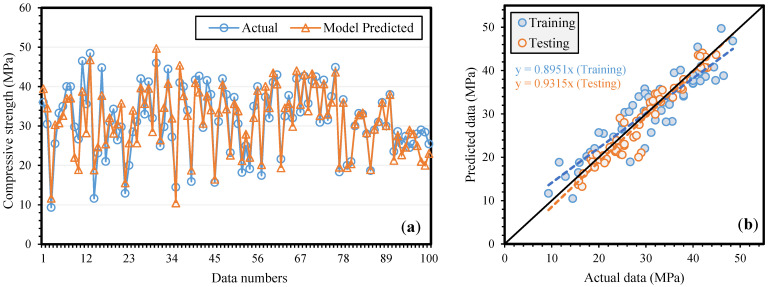
Comparison of actual and model predicted compressive strength (**a**) variation in data (**b**) scattered plot.

Figure 3a,b shows a comparison plot of experimental and projected $f_{c}^{\prime }$ along with the expression for the regression line for all three sets, i.e., training, and testing. The slope of the line is known to be exactly equal to one for an ideal situation. Figure 3 shows that the established MEP model included the influence of all five inputs and delivered a high correlation between experimental and projected results, as evidenced from the slopes of training and testing, i.e., 0.8951 and 0.9315, respectively. The graph also infers that the established model has been trained and has a high generalization relationship and thus will perform well on unseen data as well.

(10) $$f_{c}^{\prime }\;\left(\mathrm{MPa}\right)=\left(1.1x_{1}+1.1x_{2}\right)+\left(\frac{8x_{0}{}^{2}\times x_{4}}{x_{3}-x_{4}}\right){\left(16x_{0}{}^{3}\left(1.1x_{1}+1.1x_{2}\right)+\frac{4\left(5x_{1}-x_{3}\right)}{1.1x_{1}+1.1x_{2}}\right)}^{2}$$

(11) $$f_{\mathrm{STS}}\;\left(\mathrm{MPa}\right)=\left(x_{0}+\frac{x_{0}{}^{2}}{x_{0}-0.375}\right)-\left(\frac{x_{0}-0.375}{\left(x_{0}-x_{1}\right)+\left(\frac{x_{0}}{x_{0}-0.375}\right)}\right)+\frac{{\left(x_{0}-0.375\right)}^{2}}{{\left(x_{0}{}^{2}-0.375\right)}^{2}}-\left(\frac{x_{0}\times x_{1}{}^{2}}{x_{2}}\right)+\frac{x_{0}{}^{2}\times x_{3}}{x_{4}-0.375}$$

(12) $${\;f}_{\mathrm{FS}}\;\left(\mathrm{MPa}\right)=\left(\frac{2x_{3}x_{0}}{x_{4}+{\left(3x_{2}+0.97\right)}^{2}\left(2x_{1}-89x_{0}\right)}\right)+\left(\frac{2x_{0}}{\frac{\left(x_{1}-89x_{0}\right)}{\left(100x_{0}-48.5\right)}}\right)$$

where;

x0=wc; x1=BA%; x2=CC; x3=FA; x4=CA

**Figure 4:**
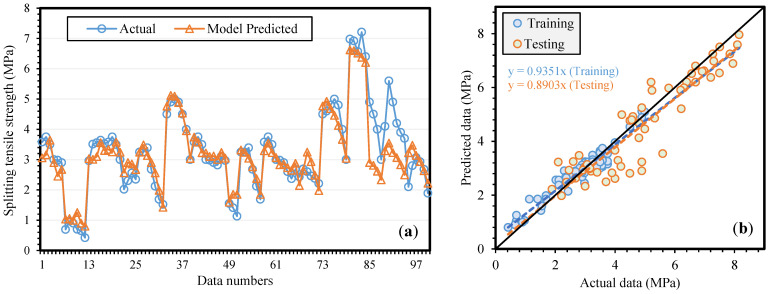
Comparison of actual and model predicted splitting tensile strength (**a**) variation in data (**b**) scattered plot.

Figure 4a,b shows a similar comparative analysis for the $f_{\mathrm{STS}}$ results. It can be observed that a good correlation exists between experimental and projected $f_{\mathrm{STS}}$. The slopes of the regression lines for the training, and testing datasets are close to ideal scenario, i.e., 0.9351, and 0.8903, respectively. The model developed for ${\;f}_{\mathrm{STS}}$ also performs extremely well on the training set. As a result, the problem of model over-fitting has been mitigated to a higher extent.

**Figure 5:**
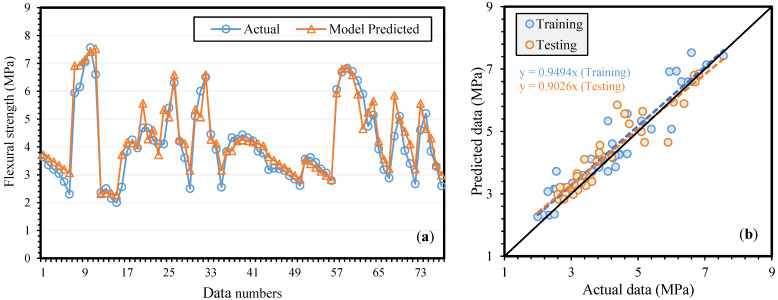
Comparison of actual and model predicted flexural strength (**a**) variation in data (**b**) scattered plot.

The graphical results of MEP model for $f_{\mathrm{FS}}$ can be observed in Figure 5a,b, which displays the regression line slope for training, and testing sets equals to 0.9494, 0.9026, respectively. It can also be observed that a better correlation between experimental and projected results was achieved for $f_{\mathrm{FS}}$ which highlighted an excellent performance of MEP on both training and testing set.

### 4.3. Models Validation by Experimental Data

**Figure 6:**
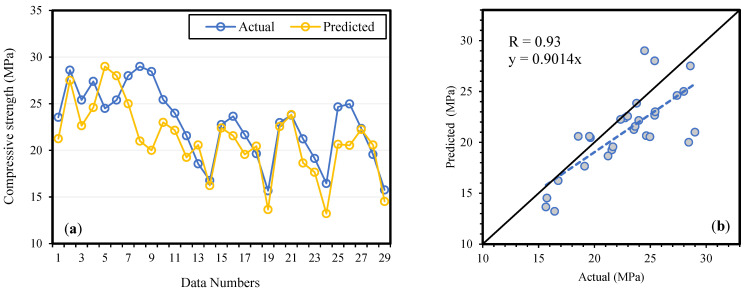
Compressive strength ($f_{c}^{\prime }$) model validation by experimental results (**a**) variation in data (**b**) scattered plot.

**Figure 7:**
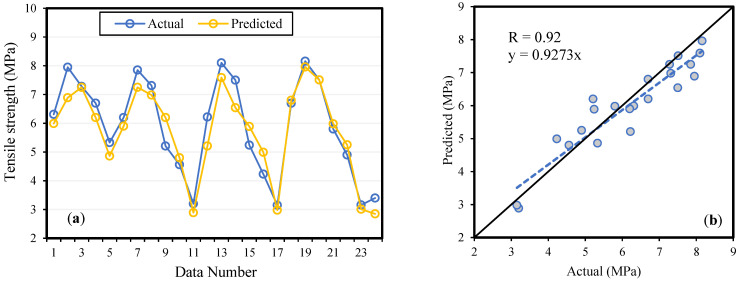
Splitting tensile strength ($f_{\mathrm{STS}}$) model validation by experimental results (**a**) variation in data (**b**) scattered plot.

**Figure 8:**
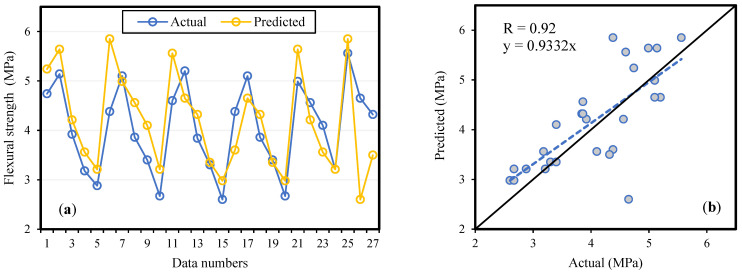
Flexural strength ($f_{\mathrm{FS}}$) model validation by experimental results (**a**) variation in data (**b**) scattered plot.

A literature survey revealed that BA concrete behaves differently at high and low replacement levels. The results of the model validation, by experimental data, are shown in Figure 6, Figure 7 and Figure 8 for $f_{c}^{\prime }\;$, ${\;f}_{\mathrm{STS}}$ and $f_{\mathrm{FS}}$, respectively. The slopes of the regression lines are 0.9014, 0.9273, and 0.9332 for $f_{c}^{\prime }$, $f_{\mathrm{STS}}$ and $f_{\mathrm{FS}}$, models which are nearly equal to 1 for the ideal case. During the models’ validation, the R value was observed to be 0.93, 0.92, and 0.92 for $f_{c}^{\prime }$, $f_{\mathrm{STS}}$ and $f_{\mathrm{FS}}$ data, respectively. The results revealed that the modeling outcome is in line with the experimental results, and the MEP model considered the effect of the parameters essential for concrete. Therefore, it has been confirmed that the ML techniques can be easily used to model the complicated processes and interaction among concrete ingredients in predicting the properties of concrete given the significant input variables.

### 4.4. Statistical Analysis and Generalizability of the Models

**Figure 9:**
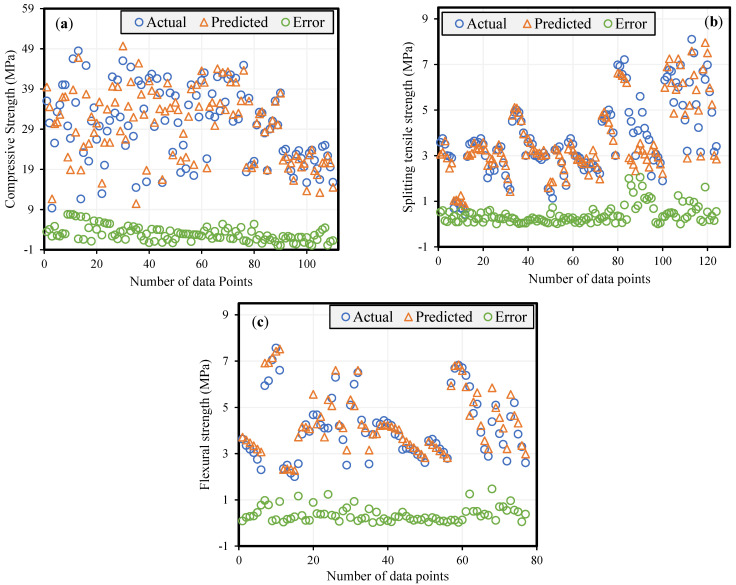
Deviation of the error between the actual and predicted results of MEP models developed for (**a**) $f_{c}^{\prime }$ (**b**) $f_{\mathrm{STS}}$ (**c**) $f_{\mathrm{FS}}$.

The amount of data points utilized for model development affects its reliability. Therefore, the ratio between data points and inputs must be higher than five for both training and testing [78]. For $f_{c}^{\prime }$, $f_{\mathrm{STS}}$ and $f_{\mathrm{FS}}$ datasets, the aforementioned ratio for the training set is 18.2, 17.5 and 17.1, respectively; and 6.4, 6.6 and 5.7, for the testing set, respectively. Moreover, Table 4 presents the outcomes of these statistical metrics for training, testing, and validation set of $f_{c}^{\prime }$, $f_{\mathrm{STS}}$ and $f_{\mathrm{FS}}$, respectively. It shows that all models have a high correlation coefficient (R) for the training set, i.e., 0.91, 0.90 and 0.91 for $f_{c\;}^{\prime }$, $f_{\mathrm{STS}}$ and $f_{\mathrm{FS}}$, respectively; and for testing set 0.94, 0.92 and 0.91, respectively. The minimum and maximum NSE for $f_{c}^{\prime }$, $f_{\mathrm{STS}}$ and $f_{\mathrm{FS}}$ models are 0.89 and 0.87; 0.91 and 0.85; and 0.86 and 0.87, respectively. The MAE and RMSE values are significantly lower for all the three sets in each model, indicating the excellent accuracy and high generalization capacity of models. The ST model can be classified as excellent based on RMSE, with values of 2.43, 2.65, and 3.25 for all three sets. Additionally, the findings show that MAE for all models is in a good range (1.45–3.98). Additionally, the OF for all three models, i.e., $f_{c}^{\prime }$ (0.036), $f_{\mathrm{STS}}$ (0.031), and $f_{\mathrm{FS}}$ (0.052) are near to zero, signifying excellent performance and thus confirming that the models successfully tackled the overfitting issue. The RRMSE values for all three developed models vary from 0.04 to 0.16, reflecting the accurateness of models. Figure 9 graphically shows the error between experimental and model predicted results in order to interpret the absolute error. The mean absolute error values are 2.87, 0.405 and 0.675 for $f_{c}^{\prime }$, $f_{\mathrm{STS}}$ and $f_{\mathrm{FS}}$, respectively. The max and min absolute errors are 1.95 and 0.075 for $f_{c}^{\prime }$, 7.76 and 0.1 for $f_{\mathrm{STS}}$, 2.15 and 0.08 for $f_{\mathrm{FS}}$, respectively.

The conditions for checking the external predictability of the MEP models are given in Table 5. The researcher proposed that one of the regression line slopes (*k* and *k’*) crossing the origin must be close to 1 [79]. Additionally, the literature has mentioned that if the indicator, *R_m_* is higher than 0.5, then the requirements for external validation of models are satisfied [80]. Table 5 shows that external validation requirements are met for all the three proposed MEP models for $f_{c}^{\prime }$, $f_{\mathrm{STS}}$ and $f_{\mathrm{FS}}$.

### 4.5. 10-Fold Cross-Validation Results

The 10-fold cross-validation can easily verify the robustness and generalized capability of ML models. This method has a parameter (k) which denotes the number of subclasses that a dataset can be split into. The 10-fold means that the given dataset can be segmented into 10 subsets or folds. This method is generally used to evaluate the ability of a model to analyze unseen data and also decreases the probability of error with random sampling.

**Figure 10:**
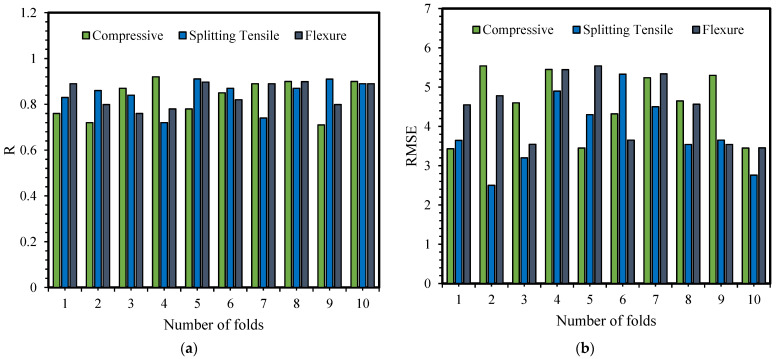
Cross-validation result of the developed models based on (**a**) R and (**b**) RMSE values.

All the three MEP models established for $f_{c}^{\prime }$, $f_{\mathrm{STS}}$ and $f_{\mathrm{FS}}$, were evaluated with 10-fold cross-validation using R and RMSE and graphically presented in Figure 10a,b, respectively. The figures show the variation in R and RMSE in each subset. However, an excellent mean accuracy can be seen. For $f_{c}^{\prime }$, $f_{\mathrm{STS}}$ and $f_{\mathrm{FS}}$, the mean value of R is 0.85, 0.89, and 0.85, respectively. In-10 fold, $f_{\mathrm{STS}}$ obtained the minimum and maximum R of 0.91 and 0 0.72, respectively. Consequently $f_{c}^{\prime }$, $f_{\mathrm{STS}}$ and $f_{\mathrm{FS}}$, achieved mean RMSE of 4.54, 3.89, and 4.78, respectively. The $f_{\mathrm{STS}}$ also has the smallest RMSE equals to 1.86, for the individual subset. Furthermore, the findings of 10-fold cross-validation demonstrate the MEP models are accurate and have a robust performance.

## 5. Conclusions

The present research implemented a twofold objective. Primarily, the mechanical properties, i.e., $f_{c}^{\prime },\;f_{\mathrm{STS}}$ and $f_{\mathrm{FS}}$ of bagasse ash concrete (BAC) were formulated by applying a supervised machine learning model, i.e., MEP. The training and testing of the models were accomplished based on widespread data collected from previous technical literature. Thereafter, sugarcane bagasse ash (BA) was used as a partial substitute for cement in various amounts (10%, 20%, 30% and 40%) to evaluate the mechanical properties. The developed MEP models were further validated through data obtained from experimental testing of BAC. The efficacy and performance of the projected models were reviewed via inferential statistical metrics, i.e., RMSE, RSE, NSE, MAE, RRMSE, ρ, OF and R. The final datasets were also cross-validated with k-fold algorithm, confirming the generalizability of models. The findings of developed models showed a good relationship with the experimental results, with R higher than 0.9; RMSE and MAE values less than 5, and OF values nearer to 0, for the all three projected MEP models for $f_{c}^{\prime }\;$, $f_{\mathrm{STS}}$ and $f_{\mathrm{FS}}$. The proposed models also met the external validation requirements found in the previous technical literature. It is clear from the current research that the consumption of bagasse ash like waste material is essential for the production of green concrete and from the sustainability viewpoint. Moreover, the MEP model, supplemented with validation on practical laboratory dataset and further cross-validation studies, can provide such models that can directly influence the civil engineering industry.

The work presented in the current research has certain shortcomings. The main focus of this research was to examine the consequence of concrete constituents on the mechanical properties of BAC. Indeed, other important factors also need to be investigated that are important to mechanical viewpoints, such as curing conditions, type of cement, reactivity and type of ash, and testing conditions. It is strongly endorsed that further research should be accomplished with an extensive dataset for model training and testing. Moreover, some deep learning techniques, i.e., convolution neural network, neuro-fuzzy inference system, and ensemble modeling, should be considered for comparative analysis and accurate assessment of concrete properties.

## Figures and Tables

**Table 1:** Best parameter setting used in MEP modeling.

Setting Parameters	Optimum Value
Subpopulation	50
length of code	40
Subpopulation size	250
Number of generations	1000
Mutation probability	0.01
Crossover probability	0.9
Mathematical operators	+, −, ×, ÷
Variables	0.5
Tournament size	4
Operators	0.5

**Table 2:** Descriptive statistics of the input variables.

Parameter	Unit	Range	Min	Max	Mean	SD
W/C	-	0.3	0.3	0.6	0.47	0.074
CC	Kg/m^3^	444	112	555	336.5	98.5
BA%	%	50	0	50	13.41	10.46
FA	Kg/m^3^	614	239	853	603.5	232.1
CA	Kg/m^3^	772	477	1249	884.6	392.3

**Table 3:** Detailed mix design proportions of the control and modified concrete.

Mix	Cement Kg/m3	CA Kg/m3	BA Kg/m3	W/C	FA Kg/m3	Water Kg/m3	Density (Kg/m3)			
							Cement	CA	FA	BA
NC	366	1013.5	0	0.5	742.3	183	3150	2510	1680	2450
10BA	329.4	1013.5	36.6	0.5	742.3	183				
20BA	292.8	1013.5	73.2	0.5	742.3	183				
30BA	256.2	1013.5	109.8	0.5	742.3	183				
40BA	219.6	1013.5	146.4	0.5	742.3	183				

**Table 4:** Inferential statistics for the training, testing, and validation datasets.

Models	Data	R	RMSE	RSE	NSE	MAE	RRMSE	ρ	OF
$f_{c}^{\prime }$	Training	0.91	3.47	0.16	0.87	2.96	0.04	0.020	
	Testing	0.94	2.98	0.12	0.89	2.98	0.09	0.046	0.036
	Validation	0.93	2.87	0.15	0.89	1.67	0.04	0.020	
$f_{\mathrm{STS}}$	Training	0.90	2.43	0.23	0.85	3.67	0.09	0.047	
	Testing	0.92	2.65	0.26	0.91	3.69	0.12	0.062	0.031
	Validation	0.92	3.25	0.31	0.90	3.98	0.10	0.052	
${\;f}_{\mathrm{FS}}$	Training	0.91	3.92	0.29	0.86	1.87	0.13	0.068	0.052
	Testing	0.91	3.34	0.28	0.87	1.45	0.15	0.078	
	Validation	0.93	3.67	0.19	0.86	2.87	0.16	0.079	

**Table 5:** Statistical indicators for verifying the external predictability of proposed MEP models.

**S.No.**	**Mathematical Expression**	**Requirement**	$\;f_{c}^{'}$	$f_{STS}$	$f_{FS}$	**Reference**
1.	$R=\frac{\sum_{i=1}^{n}\left(M_{i}-{\bar{M}}_{i}\right)(P_{i}-{\bar{P}}_{i})}{\sqrt{\sum_{i=1}^{n}{\left(M_{i}-{\bar{M}}_{i}\right)}^{2}\sum_{i=1}^{n}{\left(P_{i}-{\bar{P}}_{i}\right)}^{2}}}$	R > 0.8	0.92	0.92	0.91	[78]
2.	$k=\frac{\sum_{i=1}^{n}(M_{i}-P_{i})}{M_{i}{}^{2}}$	0.85 < k < 1.15	1.00	0.99	1.01	[79]
3.	$k^{\prime }=\frac{\sum_{i=1}^{n}(M_{i}-P_{i})}{P_{i}{}^{2}}$	0.85 < k’ < 1.15	0.98	0.98	1.05	[79]
4.	$R_{m}=R^{2}\times (1-\sqrt{\left|R^{2}-R_{0}{}^{2}\right|}$	R_m_ > 0.5	0.67	0.71	0.64	[80]
	$R_{0}{}^{2}=\frac{\sum_{i=1}^{n}{\left(P_{i}-M_{i}{}^{0}\right)}^{2}}{\sum_{i=1}^{n}{\left(P_{i}-\bar{P_{i}{}^{0}}\right)}^{2}},\;M_{i}{}^{0}=k\times P_{i}$	$R_{0}{}^{2}≅1$	0.98	0.98	0.97	
	$\overset{´}{R_{0}{}^{2}}=\frac{\sum_{i=1}^{n}{\left(M_{i}-P_{i}{}^{0}\right)}^{2}}{\sum_{i=1}^{n}{\left(M_{i}-\bar{M_{i}{}^{0}}\right)}^{2}},\;P_{i}{}^{0}=k^{\prime }\times M_{i}$	$\overset{´}{R_{0}{}^{2}}≅1$	0.98	0.99	0.98	

## Data Availability

The data used in this research was collected from published literature.

## References

[1] Du H., Dai Pang S. (2018). Value-added utilization of marine clay as cement replacement for sustainable concrete production. J. Clean. Prod..

[2] He Z., Zhu X., Wang J., Mu M., Wang Y. (2019). Comparison of CO_2_ emissions from OPC and recycled cement production. Constr. Build. Mater..

[3] Mao L.X., Hu Z., Xia J., Feng G.L., Azim I., Yang J., Liu Q.F. (2019). Multi-phase modelling of electrochemical rehabilitation for ASR and chloride affected concrete composites. Compos. Struct..

[4] Iqbal M.F., Javed M.F., Rauf M., Azim I., Ashraf M., Yang J., Liu Q.F. (2021). Sustainable utilization of foundry waste: Forecasting mechanical properties of foundry sand based concrete using multi-expression programming. Sci. Total Environ..

[5] Iqbal M.F., Liu Q.F., Azim I., Zhu X., Yang J., Javed M.F., Rauf M. (2020). Prediction of mechanical properties of green concrete incorporating waste foundry sand based on gene expression programming. J. Hazard. Mater..

[6] Frontera P., Malara A., Mistretta M., Bevilacqua C., Calabrò F., Della Spina L. (2020). Recent Trends in Sustainability Assessment of “Green Concrete”. Smart Innovation, Systems and Technologies.

[7] Pippo W.A., Luengo C.A. (2013). Sugarcane energy use: Accounting of feedstock energy considering current agro-industrial trends and their feasibility. Int. J. Energy Environ. Eng..

[8] Cordeiro G.C., Toledo Filho R.D., Fairbairn E.M., Tavares L.M., Oliveira C.H. Influence of mechanical grinding on the pozzolanic activity of residual sugarcane bagasse ash. Proceedings of the International RILEM Conference on the Use of Recycled Materials in Building and Structures.

[9] Pedersen K.H., Jensen A.D., Skjøth-Rasmussen M.S., Dam-Johansen K. (2008). A review of the interference of carbon containing fly ash with air entrainment in concrete. Prog. Energy Combust. Sci..

[10] Chusilp N., Jaturapitakkul C., Kiattikomol K. (2009). Utilization of bagasse ash as a pozzolanic material in concrete. Constr. Build. Mater..

[11] Hasan N.M.S., Sobuz H.R., Tamanna N., Slah M. (2014). Properties of concrete by using bagasse ash and recycle aggregate. Concr. Res. Lett..

[12] Jagadesh P., Ramachandramurthy A., Murugesan R. (2018). Evaluation of mechanical properties of Sugar Cane Bagasse Ash concrete. Constr. Build. Mater..

[13] Bahurudeen A., Santhanam M. Performance evaluation of sugarcane bagasse ash-based cement for durable concrete. Proceedings of the 4th International Conference on the Durability of Concrete Structures.

[14] Bahurudeen A., Wani K., Basit M.A., Santhanam M. (2016). Assesment of pozzolanic performance of sugarcane bagasse ash. J. Mater. Civ. Eng..

[15] Rerkpiboon A., Tangchirapat W., Jaturapitakkul C. (2015). Strength, chloride resistance, and expansion of concretes containing ground bagasse ash. Constr. Build. Mater..

[16] Khan M.I. (2012). Predicting properties of high performance concrete containing composite cementitious materials using artificial neural networks. Autom. Constr..

[17] Dantas A.T.A., Leite M.B., de Jesus Nagahama K. (2013). Prediction of compressive strength of concrete containing construction and demolition waste using artificial neural networks. Constr. Build. Mater..

[18] Golafshani E.M., Behnood A., Arashpour M. (2020). Predicting the compressive strength of normal and High-Performance Concretes using ANN and ANFIS hybridized with Grey Wolf Optimizer. Constr. Build. Mater..

[19] Parichatprecha R., Nimityongskul P. (2009). Analysis of durability of high performance concrete using artificial neural networks. Constr. Build. Mater..

[20] Farooq F., Nasir Amin M., Khan K., Rehan Sadiq M., Javed M.F., Aslam F., Alyousef R. (2020). A Comparative Study of Random Forest and Genetic Engineering Programming for the Prediction of Compressive Strength of High Strength Concrete (HSC). Appl. Sci..

[21] Zhang J., Li D., Wang Y. (2020). Toward intelligent construction: Prediction of mechanical properties of manufactured-sand concrete using tree-based models. J. Clean. Prod..

[22] Sun Y., Li G., Zhang J., Qian D. (2019). Prediction of the strength of rubberized concrete by an evolved random forest model. Adv. Civ. Eng..

[23] Chou J.S., Chiu C.K., Farfoura M., Al-Taharwa I. (2011). Optimizing the prediction accuracy of concrete compressive strength based on a comparison of data-mining techniques. J. Comput. Civ. Eng..

[24] Deng F., He Y., Zhou S., Yu Y., Cheng H., Wu X. (2018). Compressive strength prediction of recycled concrete based on deep learning. Constr. Build. Mater.

[25] Alexiadis A. (2019). Deep Multiphysics and Particle–Neuron Duality: A Computational Framework Coupling (Discrete) Multiphysics and Deep Learning. Appl. Sci..

[26] Ashteyat A., Obaidat Y.T., Murad Y.Z., Haddad R. (2020). Compressive strength prediction of lightweight short columns at elevated temperature using gene expression programing and artificial neural network. J. Civ. Eng. Manag..

[27] Behnood A., Golafshani E.M. (2018). Predicting the compressive strength of silica fume concrete using hybrid artificial neural network with multi-objective grey wolves. J. Clean. Prod..

[28] Sadrmomtazi A., Sobhani J., Mirgozar M. (2013). Modeling compressive strength of EPS lightweight concrete using regression, neural network and ANFIS. Constr. Build. Mater..

[29] Öztaş A., Pala M., Özbay E., Kanca E., Caglar N., Bhatti M.A. (2006). Predicting the compressive strength and slump of high strength concrete using neural network. Constr. Build. Mater..

[30] Nguyen T., Kashani A., Ngo T., Bordas S. (2019). Deep neural network with high-order neuron for the prediction of foamed concrete strength. Comput.-Aided Civ. Infrastruct. Eng..

[31] Getahun M.A., Shitote S.M., Gariy Z.C.A. (2018). Artificial neural network based modelling approach for strength prediction of concrete incorporating agricultural and construction wastes. Constr. Build. Mater..

[32] Oltean M., Groşan C., Banzhaf W., Ziegler J., Christaller T., Dittrich P., Kim J.T. (2003). Evolving evolutionary algorithms using multi expression programming. Advances in Artificial Life. ECAL 2003. Lecture Notes in Computer Science.

[33] Shah M., Alaloul W., Alqahtani A., Aldrees A., Musarat M., Javed M. (2021). Predictive Modeling Approach for Surface Water Quality: Development and Comparison of Machine Learning Models. Sustainability.

[34] Javed M.F., Farooq F., Memon S.A., Akbar A., Khan M.A., Aslam F., Alyousef R., Alabduljabbar H., Rehman S.K.U. (2020). New prediction model for the ultimate axial capacity of concrete-filled steel tubes: An evolutionary approach. Crystals.

[35] Aslam F., Farooq F., Amin M.N., Khan K., Waheed A., Akbar A., Alabdulijabbar H. (2020). Applications of Gene Expression Programming for Estimating Compressive Strength of High-Strength Concrete. Adv. Civ. Eng..

[36] Shah M.I., Memon S.A., Khan Niazi M.S., Amin M.N., Aslam F., Javed M.F. (2021). Machine Learning-Based Modeling with Optimization Algorithm for Predicting Mechanical Properties of Sustainable Concrete. Adv. Civ. Eng..

[37] Oltean M., Grosan C. (2003). A comparison of several linear genetic programming techniques. Complex Syst..

[38] Shah M.I., Amin M.N., Khan K., Niazi M.S.K., Aslam F., Alyousef R., Mosavi A. (2021). Performance Evaluation of Soft Computing for Modeling the Strength Properties of Waste Substitute Green Concrete. Sustainability.

[39] Arabshahi A., Gharaei-Moghaddam N., Tavakkolizadeh M. (2020). Development of applicable design models for concrete columns confined with aramid fiber reinforced polymer using Multi-Expression Programming. Structures.

[40] Oltean M., Dumitrescu D. (2002). Multi Expression Programming.

[41] Gandomi A.H., Faramarzifar A., Rezaee P.G., Asghari A., Talatahari S. (2015). New design equations for elastic modulus of concrete using multi expression programming. J. Civ. Eng. Manag..

[42] Sharifi S., Abrishami S., Gandomi A.H. (2020). Consolidation assessment using Multi Expression Programming. Appl. Soft Comput..

[43] Zhang Q., Yang B., Wang L., Zhu F. Predicting cement compressive strength using double-layer multi-expression Programming. Proceedings of the 2012 Fourth International Conference on Computational and Information Sciences.

[44] Alavi A.H., Mollahasani A., Gandomi A.H., Bazaz J.B. (2012). Formulation of secant and reloading soil deformation moduli using multi expression programming. Eng. Comput..

[45] Mousavi S.M., Gandomi A.H., Alavi A.H., Vesalimahmood M. (2010). Modeling of compressive strength of HPC mixes using a combined algorithm of genetic programming and orthogonal least squares. Struct. Eng. Mech..

[46] Srinivasan R., Sathiya K. (2010). Experimental study on bagasse ash in concrete. Int. J. Serv. Learn. Eng..

[47] Patel J.A., Raijiwala D. (2015). Experimental study on use of sugar cane bagasse ash in concrete by partially replacement with cement. Int. J. Innov. Res. Sci. Eng. Technol..

[48] Neeraja D., Jagan S., Kumar S., Mohan P.G. (2014). Experimental Study on Strength Properties of Concrete by Partial Replacement of Cement with Sugarcane Bagasse Ash. Nat. Environ. Pollut. Technol..

[49] Ganesan K., Rajagopal K., Thangavel K. (2007). Evaluation of bagasse ash as supplementary cementitious material. Cem. Concr. Compos..

[50] Subramani T., Prabhakaran M. (2015). Experimental study on bagasse ash in concrete. Int. J. Appl. Innov. Eng. Manag..

[51] Rukzon S., Chindaprasirt P. (2012). Utilization of bagasse ash in high-strength concrete. Mater. Des..

[52] Cordeiro G.C., Toledo Filho R.D., Tavares L.M., Fairbairn E.D.M.R. (2009). Ultrafine grinding of sugar cane bagasse ash for application as pozzolanic admixture in concrete. Cem. Concr. Res..

[53] Kumar T.S., Balaji K., Rajasekhar K. (2016). Assessment of Sorptivity and Water Absorption of Concrete with Partial Replacement of Cement by Sugarcane Bagasse Ash (SCBA) and Silica Fume. Int. J. Appl. Eng. Res..

[54] Amin N.-U. (2011). Use of bagasse ash in concrete and its impact on the strength and chloride resistivity. J. Mater. Civ. Eng..

[55] Hailu B., Dinku A. (2012). Application of sugarcane bagasse ash as a partial cement replacement material. Zede J..

[56] Mangi S.A., Jamaluddin N., Ibrahim M.W., Abdullah A.H., Awal A.S.M.A., Sohu S., Ali N. (2017). Utilization of sugarcane bagasse ash in concrete as partial replacement of cement. IOP Conf. Ser. Mater. Sci. Eng..

[57] Dhengare S.W., Raut S.P., Bandwal N.V., Khangan A. (2015). Investigation into utilization of sugarcane bagasse ash as supplementary cementitious material in concrete. Int. J..

[58] Hussein A.A.E., Shafiq N., Nuruddin M.F., Memon F.A. (2014). Compressive strength and microstructure of sugar cane bagasse ash concrete. Res. J. Appl. Sci. Eng. Technol..

[59] Reddy M.V.S., Ashalatha K., Madhuri M., Sumalatha P. (2015). Utilization of sugarcane bagasse ash (SCBA) in concrete by partial replacement of cement. IOSR J. Mech. Civ. Eng..

[60] Ganesan K., Rajagopal K., Thangavel K. (2007). Evaluation of bagasse ash as corrosion resisting admixture for carbon steel in concrete. Anti-Corros. Methods Mater..

[61] Yashwanth M.K., Raghavendra A., Kumar B.N. (2017). An experimental study on alternative cementitious materials: Bagasse ash as partial replacement for cement in structural lightweight concrete. Indian Concr. J..

[62] Shafiq N., Hussein A.A.E., Nuruddin M.F., Al Mattarneh H. (2016). Effects of sugarcane bagasse ash on the properties of concrete. Proc. Inst. Civ. Eng. -Eng. Sustain..

[63] Priya K.L., Ragupathy R. (2016). Effect of sugarcane bagasse ash on strength properties of concrete. Int. J. Res. Eng. Technol..

[64] Praveenkumar S., Sankarasubramanian G. (2019). Mechanical and durability properties of bagasse ash-blended high-performance concrete. SN Appl. Sci..

[65] Xu Q., Ji T., Gao S.J., Yang Z., Wu N. (2019). Characteristics and applications of sugar cane bagasse ash waste in cementitious materials. Materials.

[66] Arenas-Piedrahita J.C., Montes-García P., Mendoza-Rangel J.M., Calvo H.L., Valdez-Tamez P.L., Martínez-Reyes J. (2016). Mechanical and durability properties of mortars prepared with untreated sugarcane bagasse ash and untreated fly ash. Constr. Build. Mater..

[67] Cordeiro G.C., Toledo Filho R.D., Tavares L.M., Fairbairn E.M.R. (2012). Experimental characterization of binary and ternary blended-cement concretes containing ultrafine residual rice husk and sugar cane bagasse ashes. Constr. Build. Mater..

[68] Elwell D.J., Fu G. (1995). Compression Testing of Concrete: Cylinders vs. Cubes.

[69] Shah M.I., Javed M.F., Abunama T. (2021). Proposed formulation of surface water quality and modelling using gene expression, machine learning, and regression techniques. Environ. Sci. Pollut. Res..

[70] Raju K.S., Murty M.R., Rao M.V., Satapathy S.C. (2018). Support Vector Machine with k-fold cross validation model for software fault prediction. Int. J. Pure Appl. Math..

[71] Kohavi R. (1995). A study of cross-validation and bootstrap for accuracy estimation and model selection. Ijcai.

[72] Azim I., Yang J., Javed M.F., Iqbal M.F., Mahmood Z., Wang F., Liu Q.F. (2020). Prediction model for compressive arch action capacity of RC frame structures under column removal scenario using gene expression programming. Structures.

[73] Gandomi A.H., Alavi A.H., Mirzahosseini M.R., Nejad F.M. (2011). Nonlinear genetic-based models for prediction of flow number of asphalt mixtures. J. Mater. Civ. Eng..

[74] Despotovic M., Nedic V., Despotovic D., Cvetanovic S. (2016). Evaluation of empirical models for predicting monthly mean horizontal diffuse solar radiation. Renew. Sustain. Energy Rev..

[75] Akram T., Memon S.A., Obaid H. (2009). Production of low cost self-compacting concrete using bagasse ash. Constr. Build. Mater..

[76] Jagadesh P., Ramachandramurthy A., Murugesan R., Sarayu K. (2015). Micro-Analytical studies on sugar cane bagasse ash. Sadhana.

[77] Souza L.M.S.D., Fairbairn E.D.M.R., Toledo Filho R.D., Cordeiro G.C. (2014). Influence of initial CaO/SiO_2_ ratio on the hydration of rice husk ash-Ca (OH)_2_ and sugar cane bagasse ash-Ca(OH)_2_ pastes. Química Nova.

[78] Frank I.E., Todeschini R. (1994). The Data Analysis Handbook.

[79] Golbraikh A., Tropsha A. (2002). Beware of q2!. J. Mol. Graph. Model..

[80] Roy P.P., Roy K. (2008). On some aspects of variable selection for partial least squares regression models. QSAR Comb. Sci..

